# Early aggregation preceding the nucleation of insulin amyloid fibrils as monitored by small angle X-ray scattering

**DOI:** 10.1038/srep15485

**Published:** 2015-10-27

**Authors:** Eri Chatani, Rintaro Inoue, Hiroshi Imamura, Masaaki Sugiyama, Minoru Kato, Masahide Yamamoto, Koji Nishida, Toshiji Kanaya

**Affiliations:** 1 Department of Chemistry, Graduate School of Science, Kobe University, Hyogo 657-8501, Japan; 2 Research Reactor Institute, Kyoto University, Osaka 590-0494, Japan; 3 College of Pharmaceutical Science, Ritsumeikan University, Shiga 525-8577, Japan; 4 Kyoto University, Kyoto 606-8501, Japan; 5 Institute for Chemical Research, Kyoto University, Kyoto 611-0011, Japan

## Abstract

The nucleation event of amyloid fibrils is one of the most crucial processes that dictate the timing and rate of the pathology of diseases; however, information regarding how protein molecules associate to produce fibril nuclei is currently limited. In order to explore this issue in more detail, we performed time-resolved small angle X-ray scattering (SAXS) measurements on insulin fibrillation, in combination with additional multidirectional analyses of thioflavin T fluorescence, FTIR spectroscopy, light scattering, and light transmittance, during the fibrillation process of bovine insulin. SAXS monitoring revealed that insulin molecules associated into rod-like prefibrillar aggregates in the very early stage of the reaction. After the formation of these early aggregates, they appeared to further coalesce mutually to form larger clusters, and the SAXS profiles subsequently showed the further time evolution of conformational development towards mature amyloid fibrils. Distinct types of structural units in terms of shape in a nano-scale order, cross-β content, and thioflavin T fluorescence intensity were observed in a manner that was dependent on the fibrillation pathways. These results suggest the presence of diverse substructures that characterize various fibrillation pathways, and eventually, manifest polymorphisms in mature amyloid fibrils.

Amyloid fibrils are a form of protein aggregate associated with the pathology of numerous human amyloidoses and neurodegenerative disorders^1^. Regardless of the species of proteins and peptides, amyloid fibrils generally exhibit common architectural properties consisting predominantly of a microscopic cross-β structure and needle-like morphology^2^,^3^,^4^. The formation of these ordered aggregates is typically described by a nucleation-dependent polymerization mechanism in which the fibril nucleation (i.e., the generation of fibril nuclei with self-propagating ability) process acts as a rate-limiting step; no detectable amount of fibrils are formed during the lag phase, but once fibril nuclei are formed, the explosive growth of amyloid fibrils is activated due to their ability for template-dependent self-propagation^5^,^6^,^7^. Therefore, the nucleation process is considered to be a key molecular event underlying the onset and propagation of diseases and its detailed study is indispensable for therapeutic advances as well as fundamental understanding of the latent capability of proteins to form amyloid structures.

Due to the supramolecular architecture of amyloid fibrils, it is naturally assumed that the intermolecular association of proteins is in many cases associated with the generation of fibril nuclei, and the presence of many assembling species such as oligomers and protofibrils have been detected in the nucleation phase^8^,^9^,^10^. A growing number of studies have examined the relationship between oligomeric agents and cytotoxicity causing pathogenesis, with a focus on the early stages of protein aggregation. Therefore, uncovering the structural details of these species is considered to be an attractive target to provide insights into the aberrant states of protein molecules biased towards fibril nucleation. Detailed structural properties have recently been proposed with a wide variety of effective methods including solid-state NMR^11^,^12^,^13^, X-ray crystallography using microcrystals^14^, small-angle X-ray scattering (SAXS) or small-angle neutron scattering (SANS)^15^,^16^,^17^,^18^,^19^,^20^,^21^, hydrogen/deuterium (H/D) exchange monitored by mass spectrometry and solution NMR^22^, proteolytic digestion^23^, photo-induced cross-linking^24^, recognition by conformation-specific antibodies^25^, and ion mobility-mass spectrometry^26^. The emerging properties of prefibrillar aggregates through these investigations will logically be followed up with how these species contribute to the formation of fibril nuclei. However, little is known about their time-dependent changes, largely due to them being often unstable and transient properties, which hamper detailed time-resolved tracking.

Among the wide variety of established analytical techniques stated above, SAXS is one of the most useful approaches to monitor the early events that direct the formation of fibril nuclei. SAXS has been extensively used to investigate the structure, folding, and conformational dynamics of globular proteins including multidomain and multisubunit proteins in the past few decades^27^; however, its application to studies on the fibrillation process has only just been initiated and there have only been a limited number of studies reported to date^15^,^16^,^17^,^18^,^19^,^20^,^21^,^28^. The SAXS technique has been frequently used in synthetic polymers to monitor the structural organization of a wide variety of crystalline polymers during the induction period of crystallization^29^,^30^,^31^. Therefore, the application of SAXS to the amyloid fibrillation will become a powerful tool that will shed light on the molecular mechanisms by which protein aggregation is initialized and evolved in the time range of the nucleation phase.

Insulin is a hormone protein and its fibrillation has been intensively examined as one of the best models among proteins that can form amyloid fibrils readily *in vitro*^32^,^33^,^34^. The transient formation and accumulation of oligomer- and protofibril-like aggregates in the fibrillation process of insulin over the time period of the lag phase have already been reported^17^,^23^,^35^,^36^,^37^,^38^,^39^,^40^,^41^, therefore, this protein is expected to be a good model for exploring the molecular mechanisms underlying the early stage of aggregation. We recently identified a new fibrillation pathway in which amyloid fibrils were formed via a prefibrillar intermediate that appeared to play the role of a building block for mature fibrils^23^. By focusing on this pathway together with a conventional pathway, seemingly in the absence of the above prefibrillar intermediate, we performed time-resolved SAXS monitoring combined with other analytical methods such as thioflavin T fluorescence, FTIR, and light scattering techniques to observe the assembling dynamics of protein molecules, based on which we elucidated the molecular mechanisms underlying the time-dependent organization of protein molecules in insulin amyloidogenesis.

## Results

### Two distinct pathways of insulin fibril formation were dependent on the concentration of NaCl

In the present study, we selected two different pathways for the formation of insulin amyloid fibrils; one that was previously identified at a high concentration of NaCl and temperature, through which a prefibrillar intermediate species with a partial amount of cross-β structure was formed without any significant length of lag time prior to the appearance of mature fibrils^23^, while the other is a conventional pathway with a lag phase that is typically observed at a moderate concentration of NaCl. Since the protein concentration and temperature used in the present SAXS analysis (i.e., 5.0 mg/ml and 80 °C) were different from those used in our previous study (i.e., 1.0 mg/ml and 75 °C), we initially investigated the dependency of the formation pathway of insulin amyloid fibrils on NaCl concentration at 5.0 mg/ml and 80 °C by using a thioflavin T (ThT) binding assay to select an appropriate NaCl concentration for the replication of the two distinct pathways observed in our previous study.

**Figure 1:**
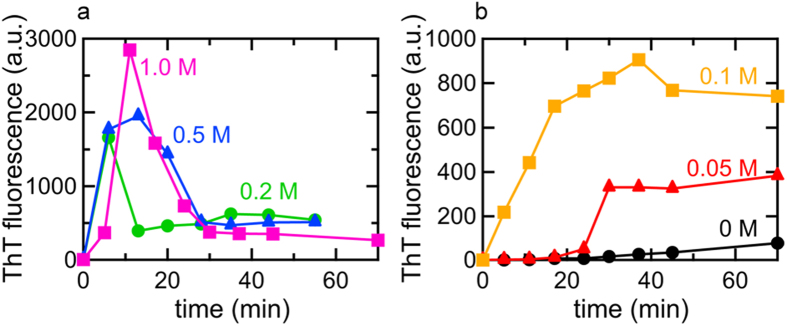
Dependence of salt concentrations on the formation pathway of insulin amyloid fibrils. The spontaneous formation of insulin amyloid fibrils was initiated by increasing the temperature to 80 °C, and time-dependent changes in ThT fluorescence intensity were then monitored. (**a**) Time courses for the formation of amyloid fibrils in the presence of 0.2 M (green), 0.5 M (blue), and 1.0 M NaCl (magenta), and (**b**) those in the absence (black), or presence of 0.05 M (red) and 0.1 M NaCl (orange) are shown.

When an insulin solution at a protein concentration of 5.0 mg/ml in the presence of various NaCl concentrations ranging from 0 M to 1.0 M was heated, a marked increase and subsequent decrease in ThT fluorescence was observed at 0.2 M NaCl and higher, suggesting the transient accumulation of prefibrillar intermediate fibrils with a high ThT fluorescence intensity in accordance with previous findings (Fig. 1a)^23^. On the other hand, a sigmoidal pattern with a lag time assigned to a typical nucleation-dependent mechanism was observed at 0.1 M NaCl and less; however, the lag time was too short to be detected on the time scale of the ThT fluorescence measurement at 0.1 M (Fig. 1b). Based on the difference observed in ThT fluorescence patterns, we focused on the conditions of 0.5 M and 0.05 M NaCl as two representative and different types of fibrillation pathways, which were further investigated by time-resolved FTIR spectroscopy to compare the formation process of cross-β structures.

**Figure 2:**
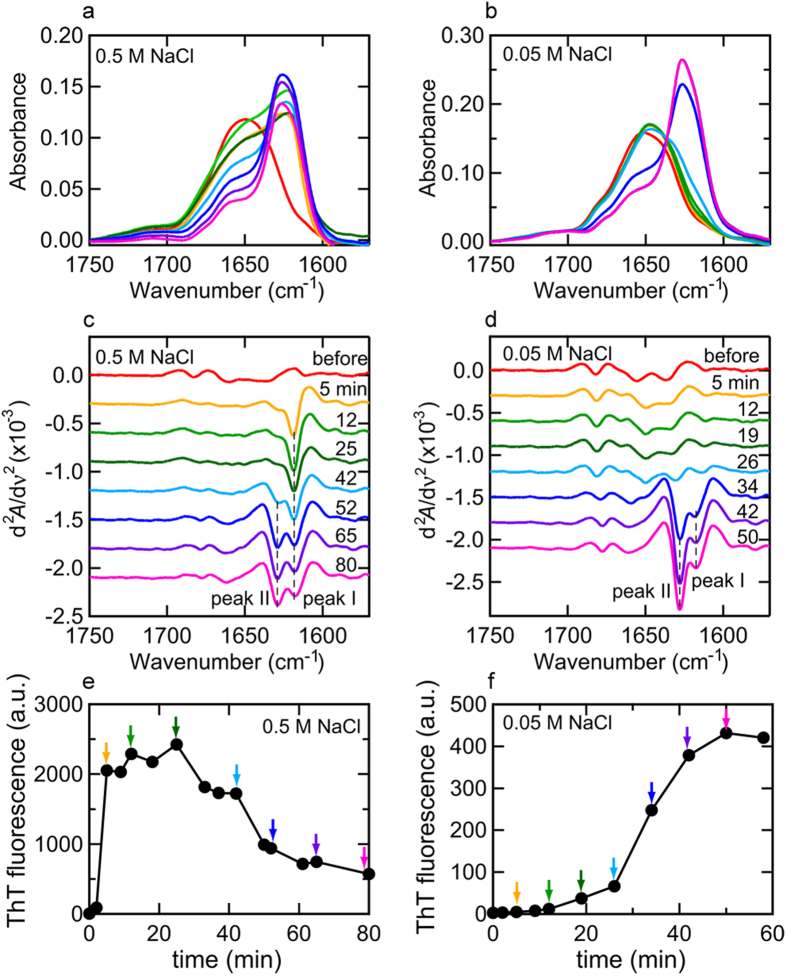
Time-dependent changes in FTIR absorption spectra at the amide I′ region during the fibrillation reaction in the presence of 0.5 M (**a**,**c**,**e**) and 0.05 M NaCl (**b**,**d**,**f**). (a,b) FTIR absorption spectra at 0.5 M (**a**) and 0.05 M NaCl (**b**). At different time points, an aliquot of the reaction solutions was placed into an optical cell and used for the FTIR measurement, which was performed at 25 °C. These measurements were performed in deuterated solution to prevent water interfering with observations of the amide I band region. In panel (**a**), the spectra monitored at 5 min (orange), 12 min (light green), 25 min (dark green), 42 min (cyan), 52 min (blue), 65 min (purple), and 80 min (magenta) after initiation of the reaction are shown, and in panel (**b**), the spectra monitored at 5 min (orange), 12 min (light green), 19 min (dark green), 26 min (cyan), 34 min (blue), 42 min (purple), and 50 min (magenta) are shown. The spectrum of insulin before heating was also represented by red lines as a reference for both conditions. Spectra were normalized so that the integrated intensity of the amide I′ band ranging from 1580 to 1750 cm^−1^ was set to be equal. (**c**,**d**) Second-derivative infrared spectra at 0.5 M (**c**) and 0.05 M NaCl (**d**). The line colors are the same as those in panels (**a**,**b**). The positions of peak I (1618 cm^−1^) and peak II (1628–1629 cm^−1^) are indicated by dashed lines. (**e**,**f**) Time course of fibrillation of FTIR samples at 0.5 M (**e**) and 0.05 M NaCl (**f**) as monitored using ThT fluorescence intensity concurrently with the FTIR analysis. The time points of sampling for the FTIR measurements are labeled by arrows with the same colors as those used in panels (**a**,**b**).

Under both conditions of NaCl, the mature fibril products formed following the completion of the reaction showed a sharp main band with a shoulder (Fig. 2a,b), which exhibited two sharp minimums at 1618 cm^−1^ and 1628–1629 cm^−1^ (peaks I and II, respectively, in Fig. 2c,d), and was consistent with the pattern typically observed for the amyloid fibrils of bovine insulin^38^,^42^. Although the rate of fibrillation appeared to be slightly slower than that observed in Fig. 1a and b for both cases at 0.5 M and 0.05 M NaCl, which was due to the deuterated solution environment in this FTIR measurement (Fig. 2e,f), the spectra obtained revealed a marked difference in the pattern of spectral development on the amide I′ region. As observed in the time-dependent changes in the values of the second derivatives at 1618 cm^−1^ and 1628 cm^−1^ (cross-β peaks I and II, respectively, in Fig. 2d), the almost coinstantaneous development of these two peaks was monitored after the lag time for 0.05 M NaCl, while the cross-β peak at 1618cm^−1^ (cross-β peak I in Fig. 2c) developed abruptly with heating and another cross-β peak at 1629 cm^−1^ (cross-β peak II in Fig. 2c) appeared thereafter for 0.5 M NaCl, supporting the formation of an immature cross-β structure characteristic to the prefibrillar intermediate^23^. On the basis of the results of the ThT and FTIR analyses, the fibrillation pathway at 0.5 M NaCl was finally selected alongside the conventional one with a lag time at 0.05 M NaCl, which were then subjected to subsequent SAXS investigations.

### Time-resolved SAXS profiles representing the formation of early aggregates as the initial stage of fibrillation

**Figure 3:**
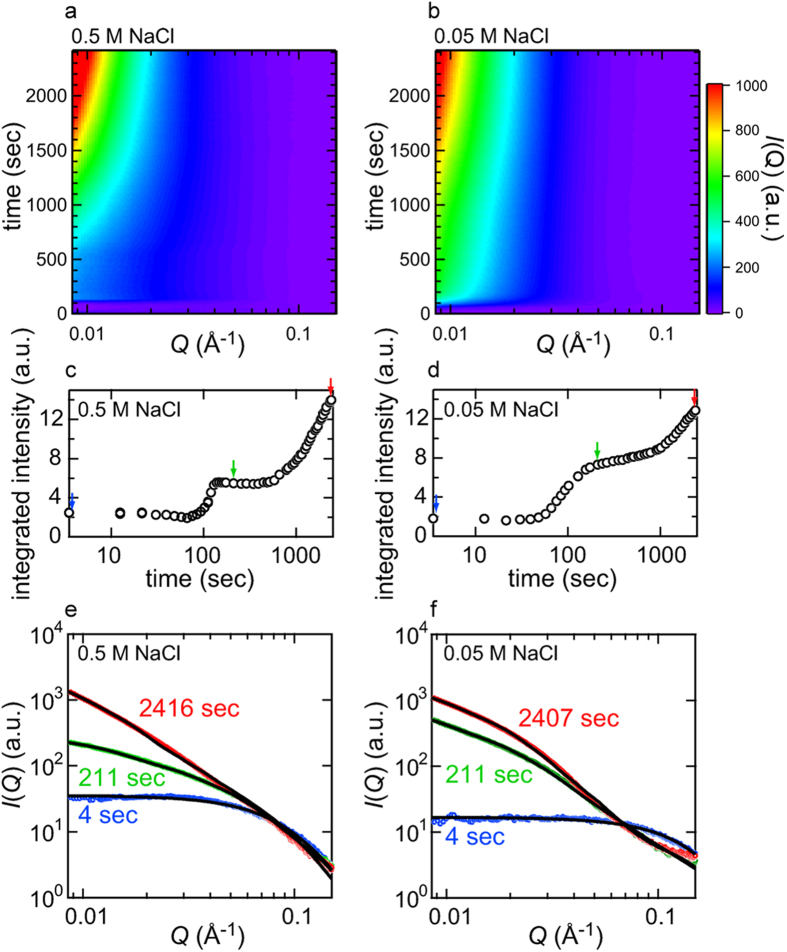
Time-dependent evolution in SAXS intensity during the fibrillation reaction observed at 0.5 M and 0.05 M NaCl. (**a**,**b**) SAXS profiles plotted against the scattering vector *Q* and reaction time as monitored at 0.5 M (**a**) and 0.05 M NaCl (**b**). In these graphs, each SAXS profile is displayed as a contour plot and stacked along the longitudinal axis representing reaction time. The scattering vector *Q* is defined as *Q *= 4πsin*θ*/*λ*, where *θ* and *λ* are a half of the scattering angle and X-ray wavelength, respectively. (**c**,**d**) Time-dependent changes in integrated SAXS intensity during the fibrillation reaction at 0.5 M (**c**) and 0.05 M NaCl (**d**). The integrated scattered intensity from 0.0085 to 0.15 Å^−1^ was plotted against reaction time to present an outline of the stepwise size development of protein aggregates. (**e**,**f**) Model fitting of SAXS profiles obtained at 4 sec (blue), 211 sec (green), and 2416 sec (red) at 0.5 M NaCl (**e**), and 4 sec (blue), 211 sec (green), and 2407 sec (red) at 0.05 M NaCl (**f**) after the initiation of the reaction. In both panels, experimental data and theoretical fitted curves are represented by colored open circles and black continuous curves, respectively. In the profiles obtained at 4 sec (blue circles in panels (**e**,**f**)), theoretical fitting was performed assuming a single cylinder, and for the profiles obtained at 211 and 2416/2407 sec (green and red circles, respectively, in panels (**e**,**f**)), fitting was performed on the basis of the two-component system with the initial state of insulin molecules before early aggregation and the aggregated species, the shape of which were assumed to be a cylinder and elliptical cylinder, respectively. More detailed overview of the fitting of the SAXS profiles are represented in Supplementary Fig. S3.

We performed *in-situ* SAXS measurements of the fibrillation reaction of insulin dissolved at a concentration of 5.0 mg/ml in 25 mM HCl containing 0.5/0.05 M NaCl, which was triggered by increasing the temperature to 80 °C. In both pathways under the two different NaCl concentration conditions, overviews of the time-dependent changes in the scattering profiles indicated a marked increase in intensity, especially at the low *Q* region, with the progression of heat-induced spontaneous fibrillation, suggesting that the SAXS profiles successfully tracked the supramolecular association of insulin molecules (Fig. 3a,b).

In both pathways, the marked development of the scattering intensity appeared to have already started approximately 100 sec after initiating the reaction and a significant increase in *I*(*Q*) was observed in the early parts of the reaction time period; however, the degree of scattering increments appeared to be more prominent at 0.05 M NaCl than at 0.5 M NaCl (Fig. 3a,b). The significant accumulation of aggregated species was unexpected at 0.05 M NaCl, because no evidence of the formation of amyloid fibrils or other aggregated species was detected in terms of ThT fluorescence intensity and FTIR spectral changes in such an early time region (Figs 1b and 2b,d).

In order to roughly evaluate changes in the profile, the integrated scattering intensity as a function of time was evaluated for each NaCl concentration. A marked increase was observed approximately 100 sec after the initiation of the reaction and then plateaued (Fig. 3c,d), where the shapes of the SAXS profiles were almost time-independent at both concentrations of NaCl (Supplementary Fig. S1). Regarding the time-dependent profile changes at 0.5 M NaCl, the time region of this step-like transition in the SAXS profile (i.e., at approximately 100 sec) appeared to correspond to that of the accumulation of prefibrillar intermediates based on the time course of ThT fluorescence intensity (Figs 1a and 3c). In time-dependent profile changes at 0.05 M NaCl, the transition of the SAXS profile occurred within the lag time of the ThT fluorescence measurement (Figs 1b and 3d). This result suggested that, at least in the case of insulin fibrillation under the conditions focused on in the present work, a protein aggregation event was initiated in the very early time period at around 100 sec regardless of the pathways of fibrillation, which we referred to as early aggregation in the present study. After the early aggregation, the integrated scattering intensity further increased in tandem with a decrease and increase in ThT fluorescence intensity at 0.5 M and 0.05 M NaCl, respectively (Figs 1a,b and 3c,d), implying further development of the aggregated structure.

### Model fitting analysis for structural characterization of early aggregates

Model fitting of the experimental profiles was attempted in order to obtain information on the structural properties of the aggregated species populating after the early aggregation as well as the initial conformational changes in insulin molecules by the temperature increase. The simplest regression was to assume the form factor of a rigid body model with a simple geometry and the constant distribution of electron density inside^43^. Regarding scattering profiles prior to the above-stated early aggregation, Nayak *et al.* previously succeeded in describing the scattering curve of the initial insulin structure with a cylindrical shape model^39^; therefore, we also attempted to adopt this model for the analysis of the initial insulin structure before temperature reached 80 °C. When a cylinder model with a radius and length of *R* and *L*, respectively, was assumed by using the following equation,


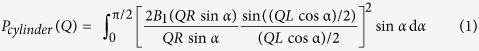


where *P*_*cylinder*_ (*Q*) and *B*_1_(*x*) are the randomly oriented cylinder form factor and the first order Bessel function, respectively^43^, a good fit was obtained for the initial profile at 4 sec (Fig. 3e,f, blue circles) with radius and height of 16 Å and 63 Å, respectively, at 0.5 M NaCl and 11 Å and 40 Å, respectively, at 0.05 M NaCl. The estimated size and shape of the cylinder were not identical between 0.5 M and 0.05 M NaCl, indicating that the initial states of insulin molecules differed in a manner that was dependent on the NaCl concentration, mainly attributed to differences in the association state and unfolded level of the native insulin. The time-dependent changes in the size and shape of the cylinder were also different between 0.5 M and 0.05 M NaCl, supporting the difference in the initial association state of native insulin molecules between the two different concentrations of NaCl (Supplementary Fig. S2).

**Figure 4:**
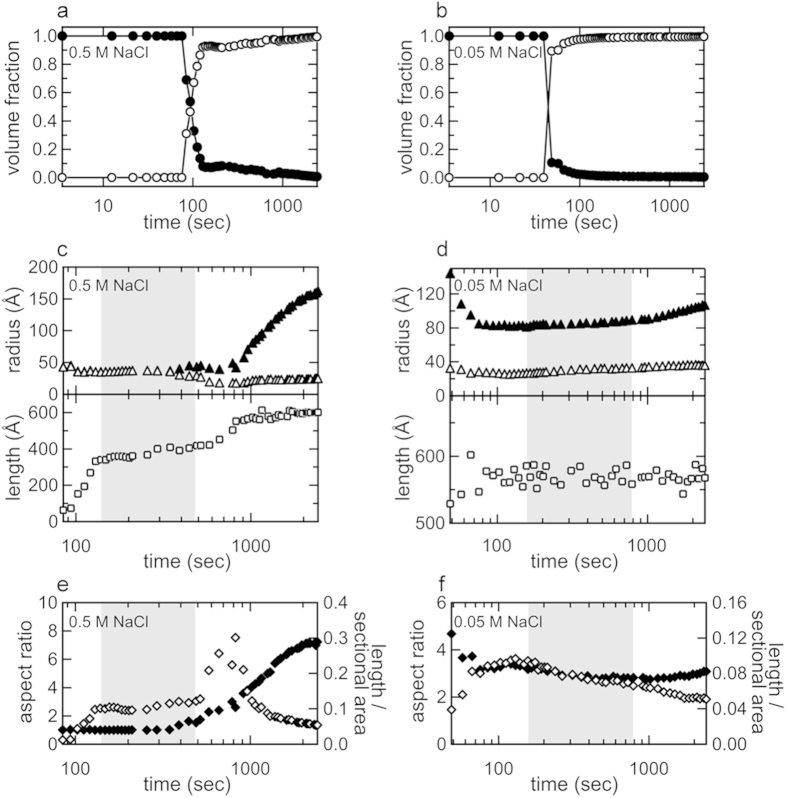
Development in the size and shape of aggregates during the fibrillation reaction as indicated by fitting of SAXS profiles. (**a**,**b**) Time-dependent changes in the volume fraction of the initial state of insulin (closed circles) and aggregates (open circles) obtained from the analysis of time-resolved SAXS measurements performed at 0.5 M (**a**) and 0.05 M (**b**). (**c**, **d**) Changes in the values of major and minor radii (closed and open triangles, respectively) and length (open squares) of the elliptical cylinder estimated at 0.5 M (**c**) and 0.05 M NaCl (**d**). (**e**, **f**) Changes in the aspect ratio of the major to minor radii (closed diamonds) and the ratio of the length to the cross-sectional area (open diamonds) of the elliptical cylinder at 0.5 M (**e**) and 0.05 M NaCl (**f**). In panels c-f, shaded regions indicate the time domains representing plateau values of integrated scattering intensity with a similar shape of SAXS scattering profiles (see Supplementary Fig. S1).

After early aggregation, the scattering profiles exhibited significant evolution, especially in the lower scattering *Q*-range (Fig. 3e,f), becoming intolerant of performing regression only with the small cylindrical shape representing the initial form, thereby supporting the formation of larger particles. Vestergaard *et al.* previously succeeded in describing the fibrillation process of insulin under different reaction conditions by analyzing experimental data with a multi-component model^17^. Based on these findings, we assumed a two-component system with an elliptical cylinder model representing newly-formed aggregates, in addition to the above-described cylinder representing the unaggregated insulin species, by using the following equation,


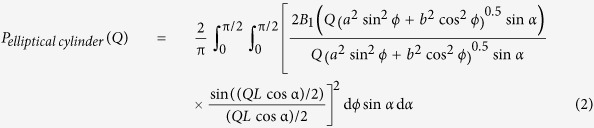


where *P*_*elliptical cylinder*_(*Q*) is the randomly oriented elliptical cylinder form factor, *a* and *b* are major and minor radii, and *L* is length^43^, and for the curve fitting, the radius and length of the cylinder were fixed to the constant values for unaggregated insulin molecules taken after the heat-induced dissociation and/or unfolding (see Supplementary Fig. S2). This two-component system reproduced the experimental patterns over the whole time scale of the measurement (Fig. 3e,f and Supplementary Fig. S3), and, as a result of plotting the volume fraction of each component against reaction time, a large amount of the unaggregated insulin species was depleted within approximately 100 sec of the start of the reaction (Fig. 4a,b), suggesting that the insulin molecules originally contained in the reaction mixture were almost fully converted to the aggregated species rapidly. When the estimated value for the major and minor radii and length of the elliptical cylinder were plotted against reaction time, the size and shape of the early aggregates were maintained for a significant length of time (Fig. 4c,d), that is suggestive of the accumulation of the early aggregates in agreement with the unchanged scattering profiles in the corresponding time regions (Supplementary Fig. S1). Additionally, a gradual lengthening of the elliptical cylinder in the axis direction was observed especially as a formation process of the early aggregates at 0.5 M NaCl, whilst this tendency was less prominent at 0.05 M NaCl (Fig. 4c–f). Following the accumulation of early aggregates, they appeared to grow again, and this may have been due to the maturation process towards needle-like fibers (Fig. 4c–f).

### Concurrent monitoring of the size development of protein aggregates at a different space scale

**Figure 5:**
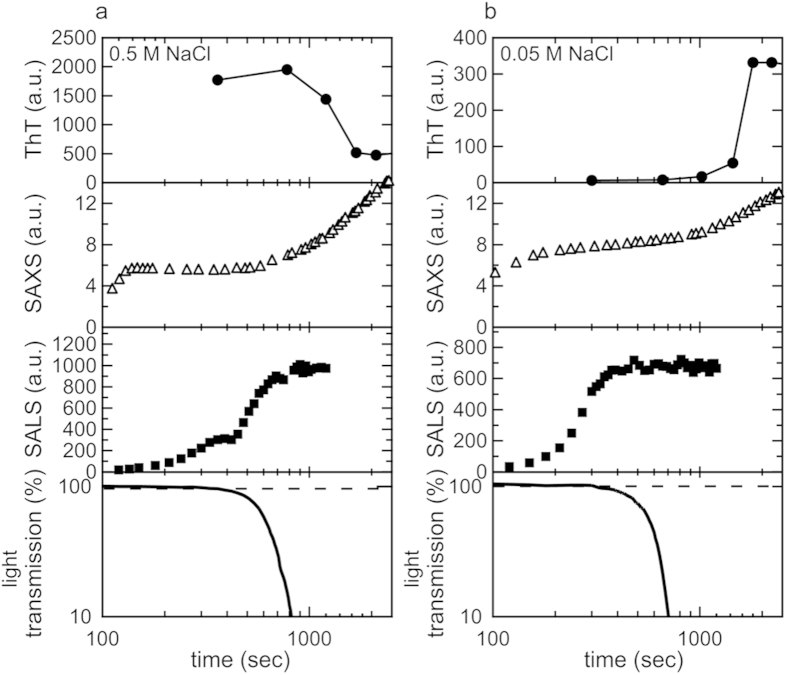
Summary of size development of the fibrillar structure monitored at different space scales. The results of time-dependent changes in the intensity of ThT fluorescence, integrated SAXS intensity, integrated SALS intensity, and light transmittance obtained at 0.5 M (**a**) and 0.05 M NaCl (**b**) are compared. ThT fluorescence data are the same as those shown in Fig. 1. Regarding the intensity of SAXS and SALS, experimental values were used for comparisons. The initial measurement value for light transmittance was set to 100%.

To further explore how the early aggregation reaction proceeded in more detail, we performed time-resolved small-angle light scattering (SALS) and light transmittance measurements, which are valuable analytical methods for tracking protein aggregation in a more macroscopic space scale than SAXS measurements. In the SALS measurement, the integrated scattering intensity between 0.2 to 5 μm^−1^ increased approximately from 200 sec following the early-stage scattering increase in the SAXS region (Fig. 5). The subsequent decline in light transmittance suggested further size development (Fig. 5). Since the size and shape of the initial aggregated species detected by SAXS remained constant over the time period with the increase in the integrated intensity of SALS and decrease in light transmittance, the early aggregated species as detected by SAXS may have served as a building block for the further construction of larger-sized clusters.

This assembling process appeared to differ between the two fibrillation pathways based on the SALS result, i.e., a two-step increase was observed at 0.5 M NaCl, whereas only one step was identified in the presence of 0.05 M NaCl (Fig. 5). Collectively, these results suggested that different processes of aggregation were responsible for the structural development of amyloid fibrils in addition to the generation of different species of prefibrillar intermediates depending on the fibrillation pathway.

### Structural investigation of the final product of insulin amyloid fibrils by SAXS, AFM, and seeding reactions

**Figure 6:**
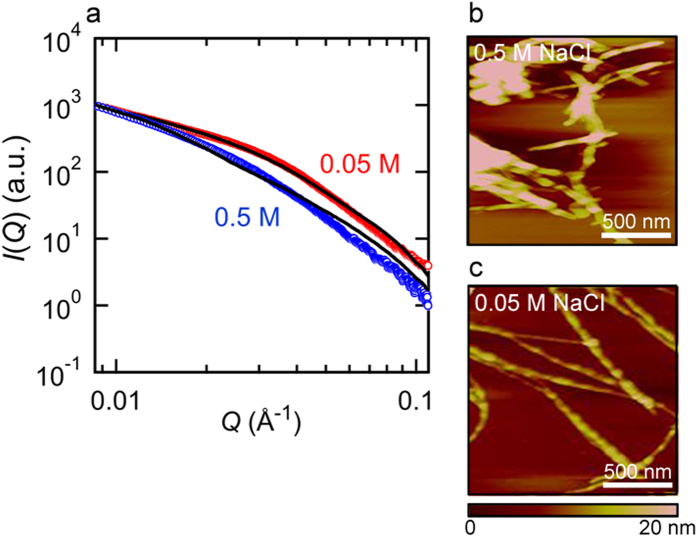
Effects of salt concentrations on the structure of final fibril products. (**a**) SAXS profiles of mature amyloid fibrils preformed at two different NaCl concentrations. This measurement was performed at room temperature. Blue and red circles represent amyloid fibrils formed in the presence of 0.5 and 0.05 M NaCl, respectively, and black lines represent theoretical fitted curves assuming a single elliptical cylinder. The major and minor radii and the length estimated from the curve fitting were 135, 22, and 635 Å, respectively, at 0.5 M NaCl and 66, 24, and 621 Å, respectively, at 0.05 M NaCl. (**b**,**c**) AFM images of the insulin amyloid fibrils formed at 0.5 (**b**) and 0.05 M NaCl (**c**). The scale bars represent 500 nm.

Based on the results of curve fitting, the shape and size of early aggregates appeared to differ in a manner that depended on the fibrillation pathway (Fig. 4c,d). Previous studies reported that solution environment or even modifications of insulin’s primary structure often induces polymorphism of amyloid fibrils^44^,^45^,^46^, and in accordance with the previous observation, the size and shape of the elliptical cylinder estimated from the scattering profiles appeared to differ between the two salt conditions at the final time point of the SAXS measurements (Fig. 4c,d). To verify the difference in their final structures, the insulin amyloid fibrils formed by prolonged incubation under the two distinct NaCl conditions were further analyzed by SAXS. In line with our expectations, the profiles obtained revealed significant differences in the scattering profiles; although the estimated sizes of the elliptical cylinder did not coincide perfectly to those at the final time point of the time-resolved SAXS measurements, they still showed a significantly larger aspect ratio at 0.5 M NaCl than that at 0.05 M NaCl, supporting the structural difference in the resulting fiber structure (Fig. 6a).

In order to explore these conformational differences in terms of morphology, we performed atomic force microscopy (AFM). In both cases of amyloid fibrils, AFM images verified the formation of typical amyloid fibrils with needle-like morphologies; however, no significant difference was observed in fibril height, except that amyloid fibrils formed in the presence of 0.5 M NaCl were more likely to associate laterally (Fig. 6b,c). Furthermore, the template-dependent self-propagation reaction using the fibril products formed at 0.5 M and 0.05 M NaCl as seeds showed different elongation rates, supporting the structural difference between these two types of fibrils (Supplementary Fig. S4).

## Discussion

By applying time-resolved SAXS measurements to the previously identified pathway for the formation of insulin amyloid fibrils at a high NaCl concentration in which mature fibrils were presumably formed via an on-pathway prefibrillar intermediate in a stepwise manner^23^, we clearly tracked size developments in protein assembly preceding fibril nucleation, thereby obtaining an insight into the detailed molecular mechanisms of fibril nucleation. The most prominent observation was the rapid generation of a metastable early aggregated species with an elliptical cylinder shape, which was attributed to the prefibrillar intermediate in light of the timing of their appearance coinciding with the marked increase in ThT fluorescence intensity (Figs 3a,c and 5a). The results obtained from the SAXS experiments revealed that the shape of the analyzed early aggregates was approximated to major and minor radii and a length of 41 ± 4, 31 ± 4, and 384 ± 27 Å, respectively, by averaging the results of 12 arbitrary scattering profiles within the time region from 139 to 490 sec (Fig. 4c). On the basis of this estimated size, the number of insulin molecules constituting the early aggregate species was roughly estimated to be approximately 220 monomers by tentatively assuming the partial specific volume of the insulin molecule in the aggregated form to be 0.73 mL/g^47^; however, a more careful analysis is needed to determine a more accurate number. In many cases of SAXS or SANS measurements, long-shaped aggregates have been reported as an initial intermediate^17^,^18^,^19^,^39^. Together with these, the observation of the rod-like early aggregates with lengthening in the axis direction during their formation, which was prominently observed at 0.5 M NaCl (Fig. 4c,d), may indicate that the tandem association of protein molecules is prone to precede the formation of the amyloidogenic conformation with propagating ability, in accordance with a previous proposal^39^.

In the conventional fibrillation pathway at 0.05 M NaCl, we anticipated that no significant aggregation would occur in contrast to that observed at 0.5 M NaCl. However, similar aggregation was detected in the early stage during the ThT-negative induction period (Fig. 3b,d and 5b), suggesting that a specific early aggregated species attributed to the prefibrillar intermediate also accumulated similar to that observed at higher concentrations of NaCl; however, the stepwise characteristics of the change in SAXS were not as clear as that observed at 0.5 M NaCl (Supplementary Fig. S1a,b). The approximate values of the major and minor radii and length obtained by averaging the results of 12 arbitrary scattering profiles within the time region from 157 to 787 sec were 84 ± 2, 29 ± 1, and 571 ± 11 Å, respectively (Fig. 4d), with the approximate number of insulin molecules constituting the early aggregate species being 660 monomers, which was markedly larger than that observed at 0.5 M NaCl. In addition to differences in size, distinct ThT fluorescence intensity and cross-β content indicated that the early aggregates bear disparate sizes and structures depending on the fibrillation pathway. Together with our present results, markedly smaller-sized prefibrillar intermediates were also reported with insulin fibrillation under different solution composition and reaction temperature^17^,^39^, suggesting that the association number of protein molecules to form the prefibrillar intermediate also varied according to the fibrillation pathway. However, the exact molecular mechanisms responsible for the less pronounced degree of aggregation, in terms of the rate of early aggregation and size of formed aggregates, at 0.5 M NaCl than that at 0.05 M NaCl in spite of the general consensus that the balance between electrostatic and hydrophobic interactions is tilted towards hydrophobic interactions, resulting in more severe protein aggregation in the presence of high salt concentrations, still remain unclear.

Whether prefibrillar intermediate species including oligomers and protofibrils work as an on-pathway intermediate by undertaking the important role of a conformational unit for the formulation of mature amyloid fibrils or an off-pathway intermediate by merely acting as a reservoir of monomeric proteins is a controversial issue when the mechanisms of amyloid formation are considered, largely because of the difficulties associated with its experimental verification^8^. In the present study, the concomitant investigation of SALS and light transmittance revealed that early aggregated species continued to pile up mutually, growing to larger clusters during the time period that the SAXS profile remained unchanged before resuming further transconformation towards mature fibrils (Fig. 5). This result is similar with the clustering of oligomers to form larger species, as reported for the fibrillation process of Aβ peptides^48^. The conformational development observed at a later stage of the fibrillation reaction seemed to occur inside the cluster of early aggregates, which is consistent with the recently proposed mechanisms of fibril nucleation from the metastable aggregated forms^8^,^26^,^49^,^50^.

The formation of amyloid fibrils is often depicted as a sort of crystallization occurring on the basis of a nucleation-dependent mechanism under supersaturated conditions^5^,^45^,^51^, and it is tempting to discuss the dynamics of protein molecules during the fibril nucleation comparatively with those of other types of inorganic or organic crystallizing components during the induction period because the nucleation phase of crystallization has been attracting increasing attention for a long time as a universal phenomenon observed in many crystalline materials. In the crystallization of silicate, the microscopic structures that emerged inside the glassy state have been proposed to act as precursors for nucleation^52^, and the transient formation of tetrahedrally coordinated water molecules, referred to as a low-density amorphous (LDA) ice structure or low-density liquid (LDL) phase, was recently demonstrated experimentally and theoretically to be a precursor structure for ice nucleation under supercooled conditions of liquid water below the homogeneous ice nucleation temperature^53^,^54^. In the case of protein crystallization, the metastable liquid-liquid phase separation of proteins to provide the dense phase of a protein solute has also been proposed to be associated with the kinetic acceleration of nucleation in some cases^55^,^56^. Although the size scale and detailed structural properties appear to vary among these orderly structures before nucleation, these reports extend to an idea that the oriented association among a substance’s molecules plays an important role in fibril nucleation. Regarding the amyloid fibril formation, the gradual formation of assembled particles with semicrystalline nanostructures was observed for insulin as protein concentrations were increased under amyloidogenic solvent conditions^57^, and a recent simulation of oligomer formation of the Aβ peptide also suggested that the structures of oligomers resembled nematic droplets^10^, which is similar to the assembled and ordered region observed in a broad spectrum of the above-described substances. The formation of prefibrillar intermediates and their subsequent coalescence, i.e., mutual gathering to form a larger cluster which precedes the later structural evolution towards mature fibrils, may be one of the typical structural states leading to the proximal condensation of protein molecules, which may enhance stochastic rearrangements towards the crystal-like protein assembly prerequisite for the generation of fibril nuclei; however, the validity of this scenario needs to be examined carefully by assessing a wide variety of proteins and fibrillation pathways.

**Figure 7:**
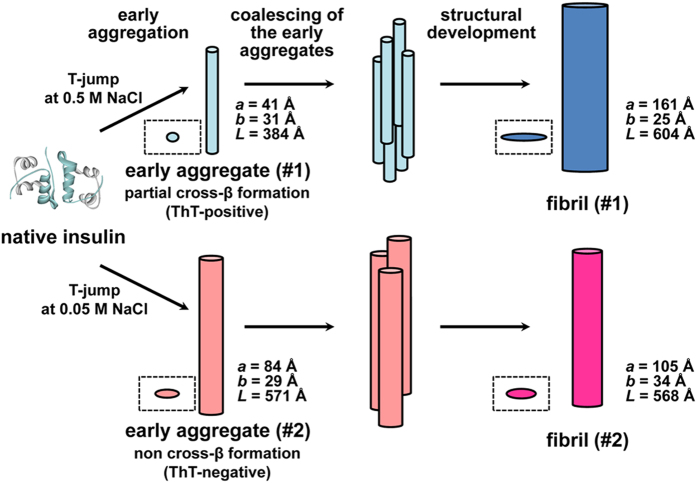
Schematic illustration representing two pathways of insulin fibrillation. With the structural illustrations for the intermediates and matured amyloid fibrils estimated from SAXS measurements, two types of fibrillation pathways at 0.5 M (upper) and 0.05 M NaCl (lower) are represented. The sizes of elliptical cylinders for the early aggregates (or prefibrillar intermediates) and the mature fibrils were obtained from the model fittings in the time range when the integrated SAXS intensity reached plateau and at the final time point of the time-resolved measurements, respectively (see Fig. 4c,d). For each elliptical cylinder species, the top-view shape is also shown in a dashed line box. It should be noted that the coalescence patterns of the early aggregates are tentatively represented and the exact number or orientation of the early aggregates species has not been determined in the present work. It should also be noted that only a single elliptical cylinder was shown for each of the fibrils as a microscopic structural unit constituting the mature amyloid fibrils.

On the basis of the present results, a schematic model for fibril organization initiated with early aggregation and its association with the formation of polymorphic amyloid structures are illustrated in Fig. 7. Our previous findings and the present results suggest that fibril formation is preceded by the accumulation of specific early aggregates that further coalesce mutually to construct larger clusters, which may promote structural development towards mature fibrils. The formation of early aggregated species may be viewed as a sort of phase separation that generates densely packed and then oriented protein domains, advantageously inducing cross-β organization. Furthermore, the discovery of the diverse features of early aggregates may facilitate a better understanding of the molecular events underlying the onset of diseases from the perspective of the cytotoxic nature of prefibrillar oligomers, which has recently been focused on as a possible pathogenic entity^8^. In light of the structural polymorphism proposed between the two different fibrillation pathways (Fig. 6), the size and structure differences in the early aggregates highlight the potential role of the early aggregation state as a determinant of final amyloid conformation dictating a specific pathway of amyloid fibrils, presumably by affecting the conformation or dynamics of critical amyloidogenic sequence regions^46^. Indeed, when time-dependent changes in the aspect ratio of the major to minor radii and the ratio of the length to the cross-sectional area of the aggregates were compared, the evolution pathways of the early aggregates towards mature fibrils appeared distinct between 0.5 M and 0.05 M NaCl (Fig. 4e,f).

The results of the present study suggest that the time-resolved monitoring of SAXS profiles and combined use of other scattering techniques with different space scales and spectroscopic methods such as thioflavin T fluorescence and FTIR spectroscopy are a powerful method for obtaining a detailed picture of the time-dependent evolution of protein aggregation coupled with the development of fibril structures. Similar approaches will be widely applicable to elucidating the structural development of the protein molecules responsible for the nucleation of amyloid fibrils. The accumulation of data for numerous species of amyloidogenic proteins will also lead to the general principles of protein aggregation governing fibril nucleation being clarified. This will be highly advantageous for identifying specific modes of amyloidogenic proteins during the induction period, which may be used in the diagnosis of amyloidoses as a unique indicator of an early sign of the proliferation of amyloid fibrils *in vivo*, and, furthermore, will provide a therapeutic indication to embody deterrent strategies, thereby contributing to the marked retardation of the onset and progression of amyloidoses and neurodegenerative disorders.

## Materials and Methods

### Heat-induced formation of insulin amyloid fibrils

The spontaneous fibrillation reaction of insulin was carried out by heating protein solutions at 80 °C. In order to measure ThT fuorescence intensity, 5.0 mg/ml bovine insulin (Sigma, St. Louis, MO) was dissolved in 25 mM HCl containing NaCl at different concentrations ranging from 0 to 1.0 M. The sample solutions were placed on a thermo-regulated heat block (Dry Thermo Unit DTU-1B; TAITEC, Nagoya, Japan), and a 7.5-μl aliquot of the sample was mixed with 1.5 ml of 5 μM ThT in 50 mM glycine-NaOH buffer (pH 8.5) at different times to examine fluorescence intensity at 485 nm with an excitation wavelength of 445 nm at room temperature^58^. Insulin concentrations were determined using an absorption coefficient of 1.0 for 1.0 mg/ml at 276 nm^42^.

Regarding FTIR measurements, 5.0 mg/ml of insulin solution was dissolved in 25 mM DCl containing 0.05 M or 0.5 M NaCl in which the amide protons of peptide groups were replaced by deuterium, and, thus, the obtained spectra exhibited amide I′ bands in a slightly shorter wavenumber range than that observed for amide I bands. At different time points, an approximately 50-μl aliquot of the sample was sealed with a cell with CaF_2_ windows and a 50-μm polytetrafluoroethylene spacer embedded inside the water-circulating system, which was thermoregulated at 25 °C. FITR spectra were then measured by collecting 256 interferograms at a resolution of 2 cm^−1^ with a FT/IR-6100 model spectrometer equipped with a DLATGS detector (Jasco, Tokyo, Japan).

### Time-resolved SAXS measurements

The real-time monitoring of fibril formation was carried out by observing a fibrillation reaction proceeding in an optical cell with a SAXS measurement at BL40B2 of SPring-8. A sample of 5.0 mg/ml of insulin dissolved in 25 mM HCl containing 0.05/0.5 M NaCl was sealed in a 1-mm path-length quartz cell embedded inside the self-produced thermoregulating system, and the temperature of the sample was increased from room temperature to 80 °C to initiate the fibrillation reaction. The temperature of the sample reached the given temperature within 60 seconds and the first SAXS profile was monitored concurrently with the temperature increase. Scattering data were then collected with an exposure time of 5 seconds at an interval of 9 seconds and a constant temperature. X-ray scattering was recorded using a CCD camera equipped with an X-ray image intensifier. The X-ray wavelength used was 1.0 Å and the camera length was 2183 mm, and the range of the scattering vector *Q* (*Q *= 4πsin*θ*/*λ*, where *θ* and *λ* are a half of the scattering angle and X-ray wavelength, respectively) in the present study was from 0.0085 to 0.15 Å^−1^. In the structural analysis of mature amyloid fibrils, the amyloid fibrils preformed by incubating 5.0 mg/ml insulin dissolved in 25 mM HCl containing 0.05/0.5 M NaCl in a microtube at 80 °C for approximately 2 hours, which was more than twice as long as the period of time required for the completion of fibrillation, were also subjected to SAXS measurements. Completion of the fibrillation reaction in the sample was confirmed on the basis of a ThT fluorescence analysis. Measurements were performed at room temperature and other conditions were fundamentally the same as those used for time-resolved SAXS measurements. The circular 1D average of the obtained image data was performed by the program FIT2D^59^.

### Atomic force microscopy

To prepare a sample plate, several microliters of fibril samples was spotted onto a freshly cleaved mica. After 1 minute, the residual solution was removed by placing a piece of filter paper at the edge of the mica plate and then dried. AFM images were obtained using a Nano Scope IIIa (Digital Instruments, Tonawanda, NY) with a scan rate of 0.5 Hz. The scanning tip used was a phosphorus (n)-doped Si (Veeco Instruments, Plainview, NY; spring constant = 20–80 N/m, resonance frequency = 245–289 kHz).

### SALS measurements

Time-resolved SALS measurements were performed with a homebuilt SALS instrument equipped with a He-Ne laser at a wavelength of 632.8 nm and a complementary metal oxide semiconductor image sensor as the light source and detector, respectively, as described previously^60^. The sample cell and thermo-regulating unit used for the SALS analysis were the same as those used for the SAXS measurements. Measurements were initiated concomitantly with increasing in the sample temperature to 80 °C and the subsequent scattering profiles were collected with an exposure time of 10 seconds at an interval of 30 seconds at a constant temperature. The camera length was 13.9 cm, resulting in the range of the scattering vector *Q* from 0.2 to 5 μm^−1^.

### Light transmission measurements

Time-resolved light transmission measurements were performed by utilizing the direct beam monitoring optics of the SALS instrument with a diode laser at a wavelength of 405 nm as the light source. The sample cell and thermo-regulating system were the same as those used for the SAXS and SALS measurements, and measurements were initiated concomitantly with increase in the sample temperature to 80 °C. Transmission was continuously measured at different time points under a constant temperature.

## Additional Information

**How to cite this article**: Chatani, E. *et al.* Early aggregation preceding the nucleation of insulin amyloid fibrils as monitored by small angle X-ray scattering. *Sci. Rep.* **5**, 15485; doi: 10.1038/srep15485 (2015).

## Supplementary Material

Supplementary Information
