# Supplementary material for: Early aggregation preceding the nucleation of insulin amyloid fibrils as monitored by small angle X-ray scattering
Source: Sci Rep. 2015 Oct 27;5:15485. doi: 10.1038/srep15485 (PMC4621412; doi:10.1038/srep15485)
Supplement: Supplementary Information [file srep15485-s1.pdf]

Supporting Information

Early aggregation preceding the nucleation of insulin amyloid fibrils as monitored by small angle X-ray scattering

Eri Chatani, Rintaro Inoue, Hiroshi Imamura, Masaaki Sugiyama, Minoru Kato, Masahide Yamamoto, Koji Nishida, and Toshiji Kanaya

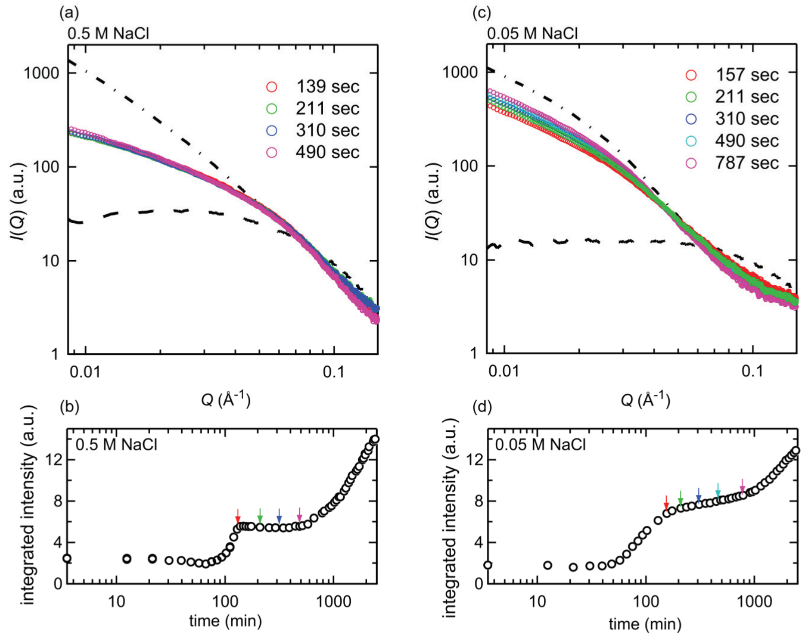

Supplementary Figure S1. SAXS profile of early aggregated species accumulating at 0.5 (a,b) and 0.05 M NaCl (c,d). (a,b) A comparison of the scattering profiles monitored at 139, 211, 310, and 490 sec in the presence of 0.5 M NaCl. As a result of overlaying the profiles, the shapes of these profiles were found to be almost identical (a), confirming that early aggregated species stably accumulated between 139 and 490 sec, corresponding to the time region exhibiting the plateau of the integrated scattering intensity (b). In panel a, dashed and dashed-dotted lines represent the scattering profiles monitored at 4 sec and 2416 sec, respectively. In panel b, the time points of the scattering profiles represented in panel a are labeled by arrows in the same colors as those used in panel a. (c,d) A comparison of the scattering profiles monitored at 157, 211, 310, 490, and 787 sec in the presence of 0.05 M NaCl. Overlaying of the profiles has showed that the shapes of these profiles were almost identical (c); however, the plateau of the time-dependent integrated scattering intensity was less prominent than that observed at 0.5 M NaCl (d). This result supports early aggregated species also accumulating, at least in the time region between 157 and 787 sec, in the conventional pathway with a significant length of the lag time. In panel a, dashed and dashed-dotted lines represent the scattering profiles monitored at 4 sec and 2407 sec, respectively. In panel d, the time points of the scattering profiles in panel c are labeled by arrows in the same colors as those used in panel c.

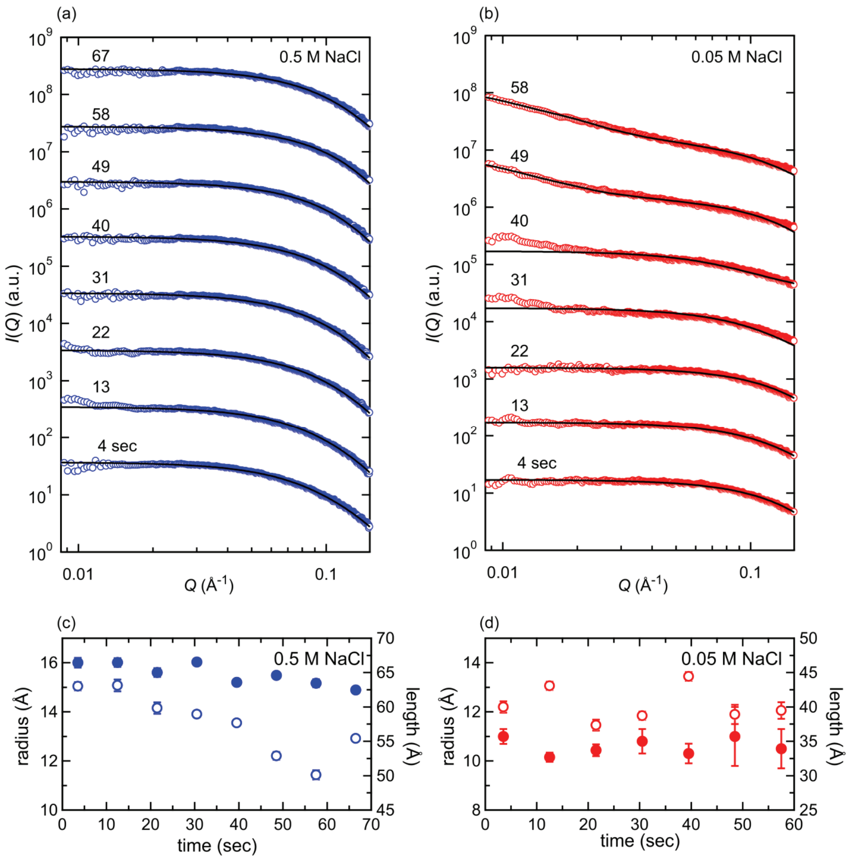

Supplementary Figure S2. Time-dependent changes in the size and shape of insulin molecules prior to the early aggregation at 0.5 M (a,c) and 0.05 M (b,d) NaCl. In order to examine conformational changes in insulin molecules during temperature increase, the size and shape of the insulin species before aggregation were estimated by performing curve fitting from 4 sec to 67 sec at 0.5 M NaCl or from 4 sec to 58 sec at 0.05 M NaCl. In panels a and b, the results of the curve fitting at 0.5 M (a) and 0.05 M NaCl (b) are shown. At 0.5 M NaCl, a good fit was obtained for the profiles from 4 to 67 sec by assuming a single cylinder shape model. At 0.05 M NaCl, on the other hand, early aggregation already started gradually from 31 sec, and the presence of the early aggregates was no longer ignorable at 49 and 58 sec; therefore, a two-component system was applied for the data analysis at 49 and 58 sec, where the sizes of both shape models were set variable. In panels c and d, the values of radius (closed circles) and length (open circles) of the cylinder shape model estimated from the above curve fittings are shown at 0.5 M (c) and 0.05 M NaCl (d). At 4 sec, the cylinder sizes were markedly different between the two different conditions of NaCl concentrations, suggesting that the insulin structure in the initial state at 0.5 M NaCl had a significantly larger size than that at 0.05 M NaCl, possibly due to the higher degree of the molecular association (c,d). As for time dependence of the cylinder size, a gradual decrease in its length was observed at 0.5 M NaCl, presumably suggesting the dissociation of insulin molecules occurring concomitantly with thermal unfolding (c). By contrast, no significant time dependency was observed at 0.05 M NaCl, implying that thermal-induced unfolding occurred without any marked effects on the dissociation of protein molecules (d).

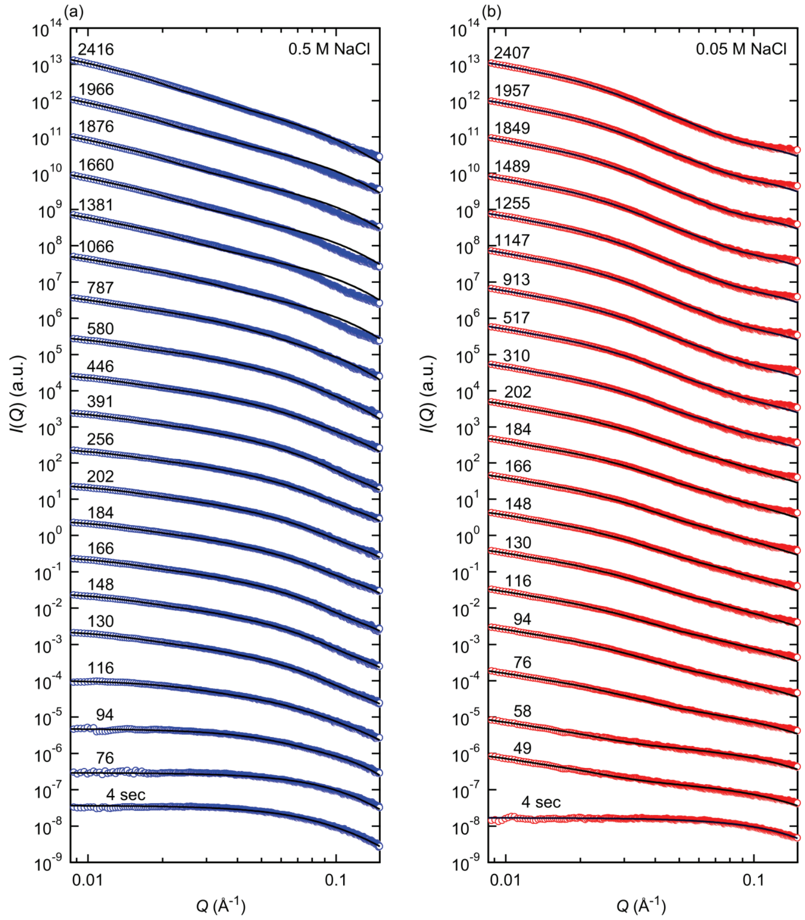

Supplementary Figure S3. Overview of fitting of the SAXS profile monitored during the fibrillation reaction at 0.5 (a) and 0.05 M NaCl (b) on the basis of the two-component system with a cylinder shape model for the initial state of insulin molecules and an elliptical cylinder shape model for the aggregates. As a form factor to represent aggregated species, we selected an elliptical cylinder in light of previous findings on rod-like intermediate species that suggested the presence of some aspect ratio for the shape of the cross-section.[1] For the curve fitting, the size of the cylinder representing the initial structure of insulin molecules was fixed to a constant value for unaggregated insulin molecules taken after the heat-induced dissociation and/or unfolding; at 0.5 M NaCl, the radius and length of the cylinder were set to be 15 Å and 53 Å, respectively, which were obtained by averaging the results at 58 and 67 sec (see Supplementary Fig. 2). At 0.05 M NaCl, since no significant change in its size was observed, the radius and height of 11 Å and 40 Å, respectively, estimated from the curve fitting at 4 sec were used.

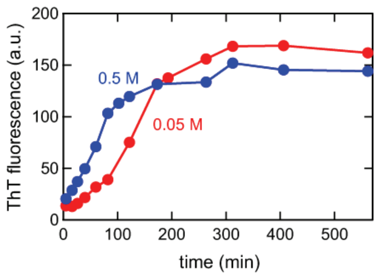

Supplementary Figure S4. Differences in seeding kinetics between amyloid fibrils formed at 0.5 M and 0.05 M NaCl. The time course of template-dependent self-propagation reaction, which is one of the most characteristic features of amyloid fibrils underlying structural polymorphisms, was investigated by adding the amyloid fibrils formed at 0.5 M and 0.05 M NaCl to a newly prepared insulin solution as seeds. Insulin was dissolved at a concentration of 0.3 mg/ml in a 25 mM HCl solution containing 100 mM NaCl in the presence of 30 μg/ml seeds formed in the presence of 0.5 M or 0.05 M NaCl. This elongation reaction was carried out in the same solvent condition in which the NaCl concentration was uniformly set to 100 mM. In order to prepare seeds, fibrils were fragmented by ultrasonication with a Microson sonicator (Misonix, Farmingdale, NY) with 20 1-s pulses at intensity level 2. The elongation reaction was carried out at 37 °C without agitation and ThT fluorescence intensity was monitored at different time points according to the same method as that used to monitor the spontaneous formation of insulin amyloid fibrils. The resulting reaction showed a different elongation rate, even under the same solvent conditions (i.e., in 25 mM HCl containing 0.1 M NaCl for both cases), supporting the structural difference between these two types of fibrils.

Reference

1 Vestergaard, B. *et al.* A helical structural nucleus is the primary elongating unit of insul in amyloid fibrils. *PLoS Biol* **5**, e134 (2007).
